# Frequency and causes of anemia in Lymphoma patients

**DOI:** 10.12669/pjms.35.1.91

**Published:** 2019

**Authors:** Tahira Yasmeen, Jamshed Ali, Khadeeja Khan, Neelam Siddiqui

**Affiliations:** 1 Dr. Tahira Yasmeen, FCPS (Medicine), Fellow Medical Oncology, Department of Medical Oncology, Shaukat Khanum Memorial Cancer Hospital and Research Centre, Lahore, Pakistan; 2 Dr. Jamshed Ali, FCPS (Medicine) FCPS (Medical Oncology), Senior Instructor, Department of Medical Oncology, Shaukat Khanum Memorial Cancer Hospital and Research Centre, Lahore, Pakistan; 3 Dr. Khadeeja Khan, MBBS, Medical Officer, Department of Medical Oncology, Shaukat Khanum Memorial Cancer Hospital and Research Centre, Lahore, Pakistan; 4 Dr. Neelam Siddiqui, MRCP, FRCP, CCST (Medical Oncology), Consultant Oncologist, Department of Medical Oncology, Shaukat Khanum Memorial Cancer Hospital and Research Centre, Lahore, Pakistan

**Keywords:** Anemia, Hodgkin lymphoma, Non Hodgkin lymphoma

## Abstract

**Objective:**

Purpose of this study was to find out frequency of anemia and its causes in newly diagnosed treatment naive lymphoma patients.

**Methods:**

We retrospectively studied all lymphoma patients (> 18 years age) diagnosed and treated at Shaukat Khanum Memorial Cancer Hospital and Research Centre, from January 2016 till January 2017. The data was collected from electronic Hospital Information System. Descriptive statistics were done by using summary measures for categorical variables as well as continuous variables.

**Results:**

Out of a total 408 patients, 272 were males and 136 females. Median age of patients was 33 years (18-76). Hodgkin lymphoma (HL) and diffuse large B cell lymphoma (DLBCL) were the diagnosis in 201 and 134 patients respectively; rest of the patients had low grade lymphomas. Anemia was present in 184 (45%) patients. Anemia of chronic disease was the commonest cause of anemia and was present in 61 (33.1%) patients. Remaining patients had anemia secondary to marrow involvement 50(27.17%); iron deficiency anemia, Vitamin B-12 deficiency anemia and hemolytic anemia were the causes in 7.6, 1.6%, % and 0.54% respectively.

**Conclusion:**

Anemia is a common feature in newly diagnosed lymphoma patients with anemia of chronic disease as the commonest cause. It is more frequent in patients with higher stages of lymphoma especially when bone marrow is involved by lymphoma. Since anemia is an important adverse prognostic factor for the outcome of lymphoma patients, work up for anemia prior to initiation of chemotherapy should be done in every lymphoma patient in order to help improve the management of these patients.

## INTRODUCTION

Lymphomas represent one of the commonest malignancies. There has been an increase in non hodgkin lymphoma (NHL) cases in past few decades and among B cell lymphomas diffuse large B cell lymphoma (DLBCL) is the commonest type.^1^ Anemia is frequently encountered in lymphoma patients and even observed before patients are started on chemotherapy and also in the absence of bone marrow involvement.^2^ It is a presenting feature in approximately 40% of patients with Hodgkin’s lymphoma (HL)^3^ and is considered an important adverse prognostic factor for outcomes of therapy^4^-^6^ especially in the background of bone marrow involvement which is yet another factor associated with poor prognosis.^7^ In patients with DLBCL anemia was found to be predictive of event free and disease free survival. It was further noted that those patients who were anemic even at six months after rituximab based therapy had higher risk for disease relapse.^8^,^9^ In addition to its association with poor prognosis in cancer patients there is correlation between levels of hemoglobin and quality of life.^10^

There are multiple factors responsible for anemia in patients with lymphoproliferative disorders, including anemia of chronic disease, iron deficiency anemia, nutritional deficiencies, autoimmune hemolytic anemia, marrow infiltration and blood loss.^11^ Several inflammatory mediators have been `identified such as interleukin 1, gamma interferon and tumor necrosis factor that inhibit erythropoiesis.^12^,^13^ Abnormal iron utilization, inappropriately low serum erythropoietin levels and decreased marrow response to erythropoietin are also responsible for anemia in these patients.^4^ In a study by Tisi MC et al. raised interleukin 6 was identified as major factor in development of anemia in patients with DLBCL.^14^ In an individual patient, more than one of these factors may be in play and responsible for anemia. There is very limited data on frequency of anemia in lymphoid malignancies in developing countries, which is very crucial to identify and treat appropriately. Aim of this study was to find out frequency and causes of anemia in lymphoma patients diagnosed over one year in our hospital.

## METHODS

Newly diagnosed treatment naïve adult (18 years and above) lymphoma patients who presented to Shaukat Khanum Memorial Cancer Hospital and Research Centre from January 2016 till January 2017 were included in this study. Lymphoma was diagnosed by histopathology from nodal or involved tissue biopsy. Medical records of all enrolled patients in study period were retrospectively analyzed. Data was collected regarding age, gender, diagnosis and stage at the time of diagnosis. Anemia was defined as hemoglobin (Hb) of < 11.5 gm/dl and it was categorized into mild (11-11.5 gm/dl), moderate (8-10.9 gm/dl) and severe (< 8 gm/dl). It was also recorded whether bone marrow involvement was present or not at time of diagnosis. Data was collected for red blood cell indices including mean corpuscular volume (MCV), mean corpuscular hemoglobin (MCH), and mean corpuscular hemoglobin concentration (MCHC). Reticulocyte count, serum iron levels, total iron binding capacity (TIBC), serum ferritin, vitamin B 12 levels, RBC folate levels were recorded. Coomb’s test was also recorded for patients in whom this investigation was done at the time of diagnosis. Reticulocyte count was categorized into < 0.5, 0.5-2 and >2. MCV, MCH, MCHC, serum iron, TIBC, serum ferritin, vitamin B 12 and RBC folate were categorized into low, normal or high except in Coomb’s test which was categorized as positive, negative or not done. All available parameters were interpreted in individual patient and cause of anemia identified accordingly. Iron deficiency anemia was defined by low ferritin, low iron and raised TIBC, while vitamin B12 and folate deficiency was ascertained by their low levels.

### Statistical Analysis

Statistical analysis was carried out using the SPSS software (version 20.0; SPSS, Chicago, IL, USA). Continuous variables were stated as Mean ± SD and categorical variables were computed as frequencies and percentages. Anemia frequency was calculated by using simple prevalence formula as (number of patients in each diagnosis / total number of cases). Patients with incomplete data were also analyzed in final analysis.

## RESULTS

The baseline description of 408 lymphoma patients with a mean age and standard deviation of 33.04 ± 11.50 years in which 197 (48.3%) were 30 years or younger (Table-I). The majority (66.7%) of participants were male with 50% diagnosed with HL. Among all patients 14.4% had stage I disease and 48.03% had stage IV disease. Anemia was present in 45% patients among which most of the anemic patients were moderately anemic (30.4%). Anemia was present in 55.97% males and 44.03% females. Anemia was observed in 53.23% HL patients and in 40.3% patients with DLBCL patients as shown in Table-II. Severity of anemia was moderate in most of anemic patients as shown in Table-III.

**Table-I:** Baseline characteristics, frequency and causes of anemia in lymphoma patients (N-408).

Variables	Frequency - N (%)	Variables	Frequency - N (%)
***Age in years***		***Extra Nodal involvement***	
18-30	197 (48.3%)	Absent	283 (69.4%)
31-40	131 (32.1%)	Present	125 (30.6%)
Above 40	80 (19.6%)	***Bone marrow involvement***	
***Sex***		Absent	306 (75.0%)
Male	272 (66.7%)	Present	83 (20.3%)
Female	136 (33.3%)	Not Done	19 (4.7%)
***Diagnosis***		***Bulky disease***	
Hodgkin Lymphoma	201 (49.3%)	Absent	247 (60.5%)
Non Hodgkin Lymphomas			
DLBCL*	134 (32.8%)	Present	161 (39.5%)
Follicular Lymphoma	18 (4.4%)	***Anemia***	
Burkitts lymphoma	16 (3.9%)		
Marginal zone lymphoma	11 (2.7%)	Absent	224 (54.9%)
Natural Killer cells	4 (1.0%)	Present	184 (45.1%)
Others#	24 (5.9%)	***Grades of Anemia***	
***Stage***		No Anemia	224 (54.9%)
I	59 (14.5%)	Mild Anemia	37 (9.1%)
II	76 (18.6%)	Moderate Anemia	124 (30.4%)
III	77 (18.9%)	Severe Anemia	23 (5.6%)
IV	196 (48.0%)	***Causes of Anemia***	
***B-Symptoms***		Anemia of chronic disease	61 (33.1%)
Absent	128 (31.4%)	Marrow involvement anemia	50 (27.17%)
Present	280 (68.6%)	Iron deficiency anemia	14 (7.6%)
***Splenomegaly***		B-12 deficiency anemia	3 (1.6%)
		Hemolytic anemia	1 (0.54%)
Absent	290 (71.1%)	Incomplete work up	55 (29.89%)
Present	118 (28.9%)		

* Diffuse large B-cell lymphoma,

# Lymphomatoid granulomatosis, Mantle cell lymphoma, Gastric MALT, anaplastic large cell lymphoma, T cell rich large B cell lymphoma.

**Table-II:** Frequency of anemia in different type of lymphomas.

Diagnosis	Prevalence
Hodgkin lymphoma	53.23%
Follicular Lymphoma	44.5%
DLBCL*	40.3%
Burkitts lymphoma	37.5%
Marginal zone lymphoma	18.2%
Natural killer cells lymphoma	25%
Others#	25%

* Diffuse large B-cell lymphoma,

# Lymphomatoid granulomatosis, Mantle cell lymphoma, Gastric MALT, Anaplastic large cell lymphoma, T cell rich large B cell lymphoma.

**Table-III:** Severity of anemia in different type of lymphomas.

Diagnosis	Mild Anemia N (%)	Moderate Anemia N (%)	Severe Anemia N (%)
Hodgkin lymphoma (201)	16 (8.0)	75 (37.3)	16 (8.0)
DLBCL*(134)	13 (9.7)	38 (28.5)	3 (2.2)
Follicular Lymphoma (18)	4 (22.2)	3 (16.7)	1 (5.6)
Burkitts lymphoma (16)	1 (6.2)	5 (31.2)	0 (0.0)
Marginal zone lymphoma (11)	2 (18.2)	0 (0.0)	0 (0.0)
Natural killer cell lymphoma (4)	0 (0.0)	1 (25.0)	0 (0.0)
Others#	1 (4.2)	2 (8.3)	3 (12.5)

* Diffuse large B-cell lymphoma

# Lymphomatoid granulomatosis, Mantle cell lymphoma, Gastric MALT, anaplastic large cell lymphoma, T cell rich large B cell lymphoma.

**Fig.1:**
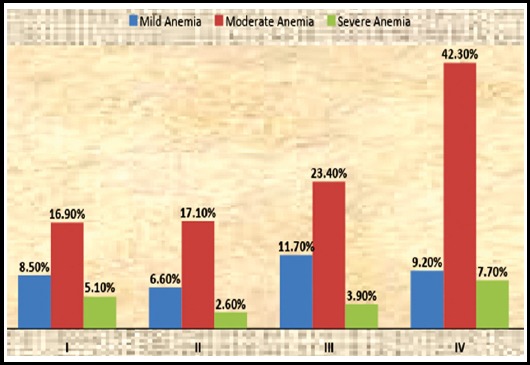
Severity of anemia in stages I-IV of lymphoma.

It was observed that anemia was more common in patients with stage IV disease (59.1% patients) with decreasing frequency in lower stages of lymphoma i.e. 38.96%, 26.3% and 30.5% in stage III, II and I respectively. Severity of anemia was moderate in all stages (Fig.1). Anemia of chronic disease was the most common cause of anemia and was present in 33.1% of patients and marrow involvement was the second common cause of anemia (27.17% patients). Iron deficiency anemia, B 12 deficiency and hemolytic anemia were the causes of anemia in 7.6%, 1.6% and 0.54% respectively in anemic patients. In 29.8% patient’s cause of anemia could not be identified due to incomplete anemia work up at baseline. Causes of anemia were analyzed separately as well in all HL and DLBCL patients. The most prevalent cause of anemia in HL and DLBCL individually was anemia of chronic disease and was present in 35 and 18 patients respectively. Anemia secondary to bone marrow involvement was the second most common cause identified in these patients and was found in 30 patients in HL and in 10 patients with DLBCL. Anemia secondary to iron deficiency was the third cause in HL and DLBCL patients.

## DISCUSSION

Anemia is commonly encountered in cancer patients; however this problem is greater in lymphoma and multiple myeloma. In patients with lymphoma, anemia has been shown to be an independent prognostic factor with worse outcome of therapy and increased mortality.^2^,^11^,^15^ Apart from its impact on survival, anemia also impairs quality of life of these patients by causing fatigue, shortness of breath, cardiovascular complications, cognitive impairment and poor performance status. Evaluation of the incidence and etiology of pretreatment anemia could help improve the prognostication and management of these patients.

According to a report by European Cancer Anemia Survey (ECAS), 39% of lymphoma patients were anemic at the time they were enrolled in the survey and only 47.3% of the anemic patients received any treatment for anemia during this survey, emphasizing the need to identify anemia at the time of diagnosis and adequately treating it in these patients.^16^,^17^ There is inadequate data regarding prevalence and etiology of anemia in lymphoma patients in our part of the world, especially Pakistan.

In this study our aim was to find out frequency and causes of anemia in newly diagnosed lymphoma patients. Out of a total of 408 lymphoma patients, almost half (45%) had anemia at presentation. Similar prevalence of anemia (42.4%) was found in one prospective study by Gosh et al from India^11^, while other studies of non-Hodgkin’s lymphoma reported their figures 32% and 35.3%.^2^,^4^ More lymphoma patients were male (66.7%) as compared to only 33.33% females. Another study from Pakistan also suggested that the prevalence of NHL was higher in males (69%) than in females (31%).^18^ Although male population was higher in number in our study but anemia was more frequent in female patients. Higher prevalence of anemia in females has also been reported in other similar studies, which likely reflects ongoing losses in young females in reproductive age group.^4^,^11^

Multiple factors may be involved at the same time in causing anemia in lymphoma patients. Anemia of chronic disease was identified as the most common cause of anemia in our patients as well as in other studies. The pathogenesis behind anemia of chronic disease is likely bone marrow erythroid hypoplasia, shortened red cell survival, decreased erythropoietin production and high inflammatory cytokine production by lymphoma cells.^2^,^19^-^21^ More than half of our patients (59.1%) with anemia had stage IV disease This correlation of anemia with higher stages of lymphoma has been reported in other studies as well.^4^,^11^ In developing countries majority of the patients are diagnosed with advanced stages of disease and this can be one factor for high prevalence of anemia, with bone marrow involvement as one of the causative factor. Interestingly our study revealed that anemia of chronic disease, anemia secondary to bone marrow involvement and iron deficiency anemia remained the major causes of anemia when HL and DLBCL patients were analyzed separately.

**Table-IV:** Severity of anemia in stages I-IV of lymphoma.

	I	II	III	IV
Mild Anemia	8.50%	6.60%	11.70%	9.20%
Moderate Anemia	16.90%	17.10%	23.40%	42.30%
Severe Anemia	5.10%	2.60%	3.90%	7.70%

### Limitations of the study

It was a retrospective study, in which the required tests to further characterize the cause of anemia were not done in some of the patients i.e. incomplete data about vitamin B12, folate, reticulocyte count and hemolytic profile. Secondly it was a single center study which limits its applicability on the entire population with lymphoma.

## CONCLUSION

The results of our study suggest that anemia is quite common in newly diagnosed lymphoma patients. The fact that anemia not only has an impact on survival and heralds poor prognosis, it is also detrimental for the quality of life of these patients. It is imperative that anemia should be identified, appropriately investigated and treated in all lymphoma patients at presentation. This simple step could ultimately help in overall better outcome in these patients.
